# Effect of Self- and Inter-Cultivar Grafting on Growth and Nutrient Content in Sweet Basil (*Ocimum basilicum* L.)

**DOI:** 10.3389/fpls.2022.921440

**Published:** 2022-07-22

**Authors:** Jason R. Hollick, Chieri Kubota

**Affiliations:** Department of Horticulture and Crop Science, The Ohio State University, Columbus, OH, United States

**Keywords:** grafting, inorganic nutrient, rootstock, scion, sweet basil, vegetable, vigor

## Abstract

Vegetable grafting has been applied to fruiting crops, yet only to a limited extent in leafy greens and herbs which may also benefit from grafting. In this study, we examined the effect of reciprocal grafting two sweet basil (*Ocimum basilicum* L.) cultivars of differing vigor on plant growth and leaf mineral nutrient concentration to test whether differences in growth exist due to grafting and whether such differences are due to altered plant nutrient status in two trials. Two cultivars ‘Nufar’ (NU), a high vigor cultivar, and ‘Dolce Fresca’ (DF), a cultivar developed for compact growth, were selected. Four grafted treatments (scion/rootstock) were created by self-grafting (NU/NU and DF/DF) and reciprocal inter-cultivar grafting (DF/NU and NU/DF). Un-grafted plants (ug-NU and ug-DF) served as controls. Following grafting, plants were grown for 26 or 21 days in a greenhouse. DF rootstocks decreased NU shoot dry mass (19–29%) and stem length (12%) compared to ug-NU in both trials, while dry root mass was reduced (28%) in the second trial. In contrast, NU rootstocks did not affect DF growth in the first trial but significantly decreased dry shoot (18%) and root (31%) mass, compared to ug-DF in the second. Concentration of most inorganic nutrients examined was affected by both rootstock and scion genotype. For NU scions, DF rootstocks resulted in significantly higher (5–29%) levels of nitrogen, phosphorus, potassium, calcium, magnesium, sulfur, copper, and zinc in leaf tissue than ug-NU. For DF scions, NU rootstocks resulted in significantly higher (7–9%) levels of potassium and phosphorus but significantly lower (11–23%) levels of magnesium, sulfur, boron, copper, and zinc when compared to ug-DF. Results of this study show that inter-cultivar grafting sweet basil using a more vigorous cultivar as a rootstock did not enhance the growth of a less vigorous scion and reduced concentrations of certain nutrients. However, grafting a vigorous scion to a less vigorous rootstock reduced shoot growth but generally increased nutrient concentrations. This suggests that changes in growth in grafted basil are not due to altered nutrient status. Further research is needed to determine specific physiological processes influencing grafted basil growth.

## Introduction

While plant grafting has been used as a means of crop propagation and trait improvement in woody crops for thousands of years, the last century has seen an increase in vegetable grafting. The earliest modern adoption of vegetable grafting occurred in Japan in the 1920’s when *Cucurbita* and later *Lagenaria* rootstocks were used to prevent watermelon (*Citrullus lanatus*) crop losses due to Fusarium wilt (Kubota et al., 2008). Grafting has since become an important horticultural tactic, widely adopted in many countries for managing soil-borne disease (Bie et al., 2017). In addition to disease management, other beneficial applications of grafting have been identified. For example, certain scion-rootstock combinations have proven more tolerant to environmental stress conditions including high (López-Marín et al., 2013) or low (e.g., Fu et al., 2021) temperature, nutrient deficiency (e.g., Liang et al., 2021), metal toxicity (e.g., Kumar et al., 2015), salinity (e.g., Estañ et al., 2005), drought (e.g., Penella et al., 2014), and flooding (e.g., Bhatt et al., 2015). Grafting has also been shown to improve the fruit quality (Kyriacou et al., 2017) as well as enhance overall plant vigor and yield in solanaceous (e.g., Leonardi and Giuffrida, 2006; Masterson et al., 2016) and cucurbit (e.g., Oztekin et al., 2011; Huang et al., 2016) crops. Vigor and yield are often positively correlated and therefore users of grafting consider vigor a desirable trait of grafted plants.

While commercial grafting has traditionally been limited to cucurbit and solanaceous crops, other crops have recently been shown to benefit from grafting. For example, globe artichoke (*Cynara cardunculus* var. *scolymus*) yield increased by grafting to *Verticillium*-resistant cardoon (*C. cardunculus* var. *altilis*) rootstocks (Ciccarese et al., 2012; Temperini et al., 2013). Similarly green beans (*Phaseolus vulgaris*) have been grafted in Portugal since 2010 for improved disease resistance and nutrient uptake (Koren et al., 2017). For leafy greens and herbs, their compact nature and short production cycle have thus far limited commercial grafting. However, brassicaceous plants, such as *Arabidopsis thaliana* have been used extensively as a model system to study transport of molecular signals and RNA through grafting experiments (Tsaballa et al., 2021). Additionally, agricultural applications of brassica grafting have been investigated. For example, cabbage (*Brassica oleracea* var. *capitata*) vernalization requirement was removed with radish (*Raphanus sativus* var. *caudatus*) rootstocks allowing for more efficient breeding (Motoki et al., 2019). Chen et al. (2019) reported cabbage grafted to Chinese kale (*Brassica oleracea* var. *alboglabra*) had improved head quality. This research in crops that are not traditionally grafted indicate that there are many additional species that could benefit from grafting.

Sweet basil (*Ocimum basilicum* L.) is a leafy herb from the family Lamiaceae grown worldwide in a variety of production systems ranging from open field to hydroponic greenhouse and indoor vertical production (Makri and Kintzios, 2008; Walters et al., 2020). Basil produces aromatic phenylpropanoid and terpenoid compounds that give it many culinary, cosmetic, and medicinal uses (Simon et al., 1999; Makri and Kintzios, 2008). Sweet basil is also often grown as an ornamental in home gardens and landscapes due to a wide range of phenotypes (Simon et al., 1999). Grafted basil, resembling a small tree, with a tall woody rootstock and compact scion (Hishtil Ltd., Ashkelon, Israel) has been marketed to home gardeners. However, no other commercial use of basil grafting is known and there is, to our knowledge, no previous research on the practice. Nevertheless, sweet basil cultivars differ greatly in terms of vegetative production and chemical profile (Simon et al., 1999), thus it is possible that grafting different sweet basil cultivars may create altered phenotypes beneficial to home garden or commercial production such as increases in vigor and yield similar to those observed in other crops. Even in commonly grafted and well-studied crops such as cucurbits and tomatoes, grafting-induced vigor has not been fully understood. Among limited information, enhanced water and nutrient uptake and use efficiency by grafting have been reported in cucurbits (Nawaz et al., 2017; Liang et al., 2021) and solanaceous crops (Leonardi and Giuffrida, 2006; Albornoz et al., 2018). This enhanced uptake is often attributed to improved root system size and architecture (Huang et al., 2016; Suchoff et al., 2018; Liang et al., 2021) or more efficient nutrient transporters (Albornoz et al., 2018).

The role of nutrient supply on growth and yield of sweet basil is unclear. For example, some studies with synthetic and/or organic fertilizer sources have shown increased herbage yields with nutrient supplementation (Zheljazkov et al., 2008; Pandey et al., 2016) and others reported relative insensitivity to nutrient supply (Walters and Currey, 2018). Determining ideal fertilization for basil is further complicated by the fact that growth responses to nutrient supply are often cultivar specific (Ciriello et al., 2020). Furthermore, mycorrhizal fungus *Gigaspora rosea* has been found to increase root branching, number of root tips, dry shoot mass and stem length in basil cultivar ‘Genovese’ (Copetta et al., 2006). Similarly, Burducea et al. (2018) found that treatments with a commercial mix of arbuscular mycorrhizal fungi, which are known to improve nutrient uptake in many plant species, resulted in increased plant height and shoot biomass production in one of two cultivars of basil examined. These studies suggest that modifications to the root traits and nutrient uptake can lead to improved vigor in basil. Thus, grafting could represent another means of modifying the basil root system and resulting water and nutrient uptake.

Given the wide range of diversity in sweet basil growth it is possible that grafted basil will display altered growth due to differences in nutrient uptake. For instance, a vigorous cultivar of basil used as a rootstock for a less vigorous cultivar may result in increased growth due to improved nutrient uptake, while the reciprocal combination may result in reduced growth due to impaired nutrient supply. In this study we examined the effect of reciprocal grafting two basil cultivars of differing vigor on plant growth and leaf mineral nutrient concentration to test whether differences in growth exist due to grafting and whether such differences are due to altered plant nutrient status.

## Materials and Methods

### Experimental Timeframe and Location

Two trials were conducted with the first trial running from 30 September to 9 December 2020 and the second from 15 January to 11 March 2021. Both trials were conducted in a glass greenhouse located at The Ohio State University, Columbus, Ohio (40°00′07.2′ N, 83°01′42.7′ W). Greenhouse environmental conditions were monitored using T-type thermocouples (gauge 36, connected to the extension of gauge 24) for air temperature and LI-90R quantum sensor (LI-COR, Lincoln, NE, United States) for photosynthetic photon flux density (PPFD). All sensors were calibrated properly prior to the experiment and connected to a datalogger (CR23X, Campbell Scientific, Logan, UT, United States).

### Plant Materials and Pre-transplant Growing Conditions

Two basil cultivars Dolce Fresca (Territorial Seed Company, Cottage Grove, OR, United States), a cultivar developed for compact growth, and Nufar (Johnny’s Selected Seeds, Waterville, ME, United States), a vigorous and Fusarium wilt-resistant cultivar., were used. Seeds were sown in 98 cell count plug trays (Hummert International, Earth City, MO, United States) filled with soilless potting mix (BM1; Berger Peat Moss Ltd., Saint-Modeste, QC, Canada) and gently pressed into the moistened substrate. Un-grafted control plants were sown 7 days after plants used for grafting. Seeded flats were covered with plastic trays (Hummert International, Earth City, MO, United States), to maintain humidity and placed in a glass greenhouse with temperature setpoints of 24/19°C day/night and a supplemental daily light integral (DLI) of 3.6 mol m^−2^ d^−1^ provided by overhead lighting (1,000 W metal halide lamps; PARsource, Petaluma, CA, United States). Top cover trays were removed, when approximately 85% of seedlings had visibly emerged. Seedlings were sub irrigated with water as needed, until 20 days after seeding at which point a nutrient solution (electrical conductivity: 1.6 dS m^−1^) based on a commercial fertilizer mix (Jack’s 12–4-16 Hydro FeEd RO, JR Peters Inc., Allentown, PA, United States) containing 100 mg l^−1^ nitrogen (NO_3_-N), 3.1 mg l^−1^ nitrogen (NH_4_-N), 34.2 mg l^−1^ P, 134.3 mg l^−1^ K, 59.1 mg l^−1^ Ca, 17.1 mg l^−1^ Mg, 1.25 mg l^−1^ Fe, 0.50 mg l^−1^ Mn, 0.01 mg l^−1^ Mo, 0.16 mg l^−1^ Cu, 0.30 mg l^−1^ Zn, and 0.15 mg l^−1^ B was used. The municipal source water used in this study, typically contains low levels of Ca (<30 mg l^−1^), Cl (<30 mg l^−1^), NO_3_-N (<3 mg l^−1^), Mg (<8 mg l^−1^), S (<20 mg l^−1^), K (<5 mg l^−1^), P (<0.5 mg l^−1^), Zn (<0.5 mg l^−1^), Na (<20 mg l^−1^) and Al (<0.2 mg l^−1^). Alkalinity is typically <42.0 CaCO_3_ mg l^−1^ and approximate EC is 0.2 to 0.3 dS m^−1^. During pre-transplant growth, daytime/nighttime temperatures were (means ± S.D.) 22.4 ± 0.06/17.6 ± 0.04°C (Trial 1) and 22.1 ± 0.10/18.3 ± 0.06°C (Trial 2). Total DLI (solar and supplemental light) was recorded as 11.4 ± 0.55 mol m^−2^ d^−1^ (Trial 1) and 10.9 ± 0.63 mol m^−2^ d^−1^ (Trial 2).

### Grafting Treatments

Plants were grafted at 36 and 26 days after sowing in the first and second trials, respectively. At grafting, plants exhibited two to three pairs of true leaves exceeding 1 cm in length and had stem diameters between 1.6 and 2.0 mm at 1 cm below the cotyledons. Four grafted treatments were created: (1) ‘Nufar’ self-grafted (NU/NU), (2) ‘Dolce Fresca’ self-grafted (DF/DF), (3) ‘Dolce Fresca’ scion grafted to ‘Nufar’ rootstock (DF/NU), and (4) ‘Nufar’ scion grafted to ‘Dolce Fresca’ rootstock (NU/DF). For inter-cultivar grafts (NU/DF and DF/NU), rootstocks and scions with similar stem diameter were selected; and tube grafted using 30° angles and secured with a 1.5 mm silicon grafting clip. Scions and rootstocks were cut below the cotyledons. For self-grafted plants (NU/NU and DF/DF), a single plant was cut below the cotyledons, and the upper and lower pieces rejoined. Throughout the grafting process, the plants were continuously misted with water using a hand spray bottle, to avoid desiccation. As grafts were completed, plants were set in a new 98-cell tray inside a sealed clear polycarbonate healing chamber (69 l volume, 45.7 × 66.0 × 22.9 cm; Rubbermaid, Atlanta, GA, United States with 8 cm of water in the bottom). Sealed boxes were placed in a growth chamber (Controlled Environments Ltd., Winnipeg, MB, Canada). Throughout the healing process, the temperature inside the clear box was maintained at 28°C. After 24 h of complete darkness, continuous light was applied at 60 μmol m^−2^ s^−1^ PPFD. After 5 days, the lid of the healing chamber was cracked open slightly for 48 h before plants were removed.

### Post Grafting Growing Conditions and Data Collection

After plants were removed from the graft healing chamber, plants were grown in the same greenhouse under the same lighting and temperature set points used during pre-transplant growth. After a 24-h acclimation period, plants were considered successfully grafted if the scion and rootstock remained connected after gently pulling in opposite directions. After testing graft union formation, 24 uniform, plants of each grafted treatment (NU/NU, DF/DF, NU/DF, and DF/NU) as well as each un-grafted treatment (ug-NU and ug-DF) of a similar developmental stage were transplanted into 10 × 10 × 10 cm plastic pots filled with BM1 all-purpose soilless potting mix (Berger Peat Moss Ltd., Saint-Modeste, QC, Canada). Pots were arranged in a randomized complete block design consisting of six blocks with each block containing 4 plants of each treatment. Pots were arranged in staggered rows, so that flat sides were 10-cm apart to achieve a plant density of 50 plants per square meter. Plants were fertigated with the same nutrient solution used for pre-transplant stage using drip irrigation with pressure compensated emitters (2 lh^−1^, Netafim Ltd., Hatzerim, Israel) programmed to run for 1 min 4–8 times daily to ensure water drained from pots at each irrigation event. Daytime/nighttime temperatures were 22.4 ± 0.08/18.8 ± 0.05°C (Trial 1) and 22.5 ± 0.04/18.4 ± 0.01°C inside the greenhouse. Total DLI (solar and supplemental light) was recorded as 8.7 ± 0.50 mol m^−2^ d^−1^ (Trial 1) and 16.6 ± 0.97 mol m^−2^ d^−1^ (Trial 2). When plants had 7 pairs of true leaves with a lamina length exceeding 3 cm on the main stem (26 days after transplanting (DAT) in trial 1 and 21 DAT in trial 2), two plants per treatment were randomly selected from each block (12 plants per treatment) and used for whole plant growth analyses. Plant growth analyses included shoot and root dry mass, number of leaves with lamina length exceeding 1 cm, and main stem lengths. Roots were cleaned by gentle shaking followed by washing in tubs of tap water. All shoot tissue and root tissue were separated and dried for 6 days at 55°C at which point root and shoot dry mass was determined. Shoot-to-root ratios for dry mass was determined by dividing shoot dry mass by root dry mass. Tissue nutrient analysis was conducted using dried leaves harvested at 21 DAT in the second experiment. Analysis for inorganic nutrients (N, P, K, Ca, Mg, S, Fe, Mn, B, Cu, Zn, and Na) was conducted by JR Peters Inc. (Allentown, PA, United States). Specifically, there were two sets of analyses conducted for each sample. N% was determined using a CNS Instrument (Flash EA1112, Thermo Fisher, Waltham, MA, United States) following the procedure described in AOAC (2006). All other nutrients were determined using by ICP-optical emission spectrometry (ICP-OES; Prodigy XP, Teledyne Leeman Labs, Hudson, NH, United States), after the samples were prepared according to the method adapted from Miller (1998).

### Statistical Analysis

All statistical analysis was conducted using R 4.1.2 (R Foundation, Vienna, Austria), and RStudio 1.3.1073 (RStudio PBC, Boston, MA). Main factor (scion and rootstock cultivar) effects and interactions for plant growth parameters and plant inorganic nutrient concentration in grafted plants (NU/NU, NU/DF, DF/DF, and DF/NU) were assessed by analysis of variance. Tukey’s HSD (honestly significant difference) tests were conducted at *p* = 0.05 and used for separation of means for grafted treatments. Scion effects refer to comparisons between grafted plants with NU scions (NU/NU and NU/DF) and those with DF scions (DF/DF and DF/NU) while rootstock effects refer to comparisons between grafted plants with NU rootstocks (NU/NU and DF/NU) and those with DF rootstocks (DF/DF and NU/DF). Additionally, Dunnett’s test at *p* = 0.05 was applied for comparisons of means of self- and inter-cultivar grafted plants of each cultivar to their respective un-grafted control (NU/NU and NU/DF compared to ug-NU; DF/DF and DF/NU compared to ug-DF).

## Results

### Plant Growth

**Figure 1:**
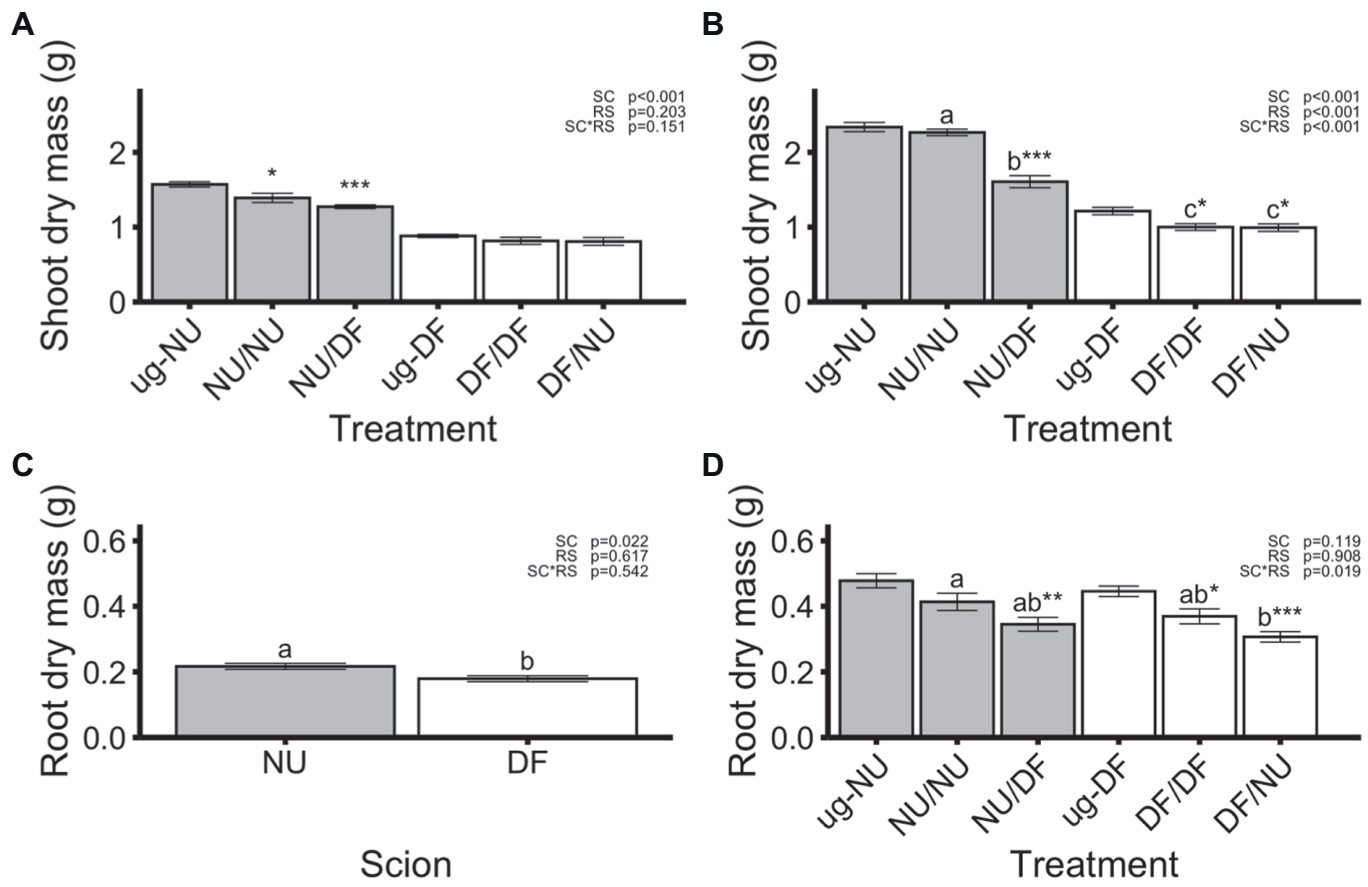
Shoot dry mass in the first **(A)** and second **(B)** trial, root dry mass in the first **(C)** and second **(D)** trial. Values are means of 6 replicates for each treatment and error bars represent standard error. Asterisks above bars indicate significant difference between grafted treatment and the un-grafted control of the same scion cultivar by Dunnett’s test [^***^(*p* ≤ 0.001), ^**^(*p* ≤ 0.01), and ^*^(*p* ≤ 0.05)]. *p*-values indicate significance of scion (SC), rootstock (RS) and scion by rootstock interaction (SCxRS) effects from the factorial analysis of self- and inter-cultivar grafted plants (four treatments excluding un-grafted controls). Letters indicate mean separations for the four grafted treatments by Tukey’s HSD (*p* ≤ 0.05) applied when SCxRS interaction was significant. Gray and white bars represent plants with NU and DF shoot systems, respectively.

Significant treatment by trial interactions were detected for leaf number, shoot mass, and root mass, thus these variables are presented separately for each trial. For the other variables, data of the two trials were combined for analysis. In the first trial, dry shoot mass (Figure 1A) was affected by scion genotype, but not rootstock nor scion x rootstock interaction, with NU scions (NU/NU and NU/DF) producing 64% greater dry mass than DF scions (DF/DF and DF/NU), regardless of rootstock genotype. Compared to un-grafted NU (ug-NU), self-grafting NU (NU/NU) and grafting NU to DF rootstocks (NU/DF) significantly reduced shoot dry mass by 12 and 19%, respectively (Figure 1A). In contrast, when DF was scion, neither self- (DF/DF) nor inter-cultivar (DF/NU) grafting significantly affected shoot mass, compared to un-grafted DF (ug-DF). In the second trial, dry shoot mass was significantly affected by scion genotype, rootstock genotype, and their interaction (Figure 1B). Both treatments with NU scions had greater dry shoot mass (+127% +61% in NU/NU and NU/DF, respectively) than treatments with DF scions (DF/DF, DF/NU). However, Grafting NU scions to DF rootstocks (NU/DF) significantly reduced dry shoot mass by 31% compared to the ug-NU. In contrast, dry shoot mass of self- (DF/DF) and inter-cultivar (DF/NU) grafted DF was similar but 18% lower than that of ug-DF (Figure 1B).

**Figure 2:**
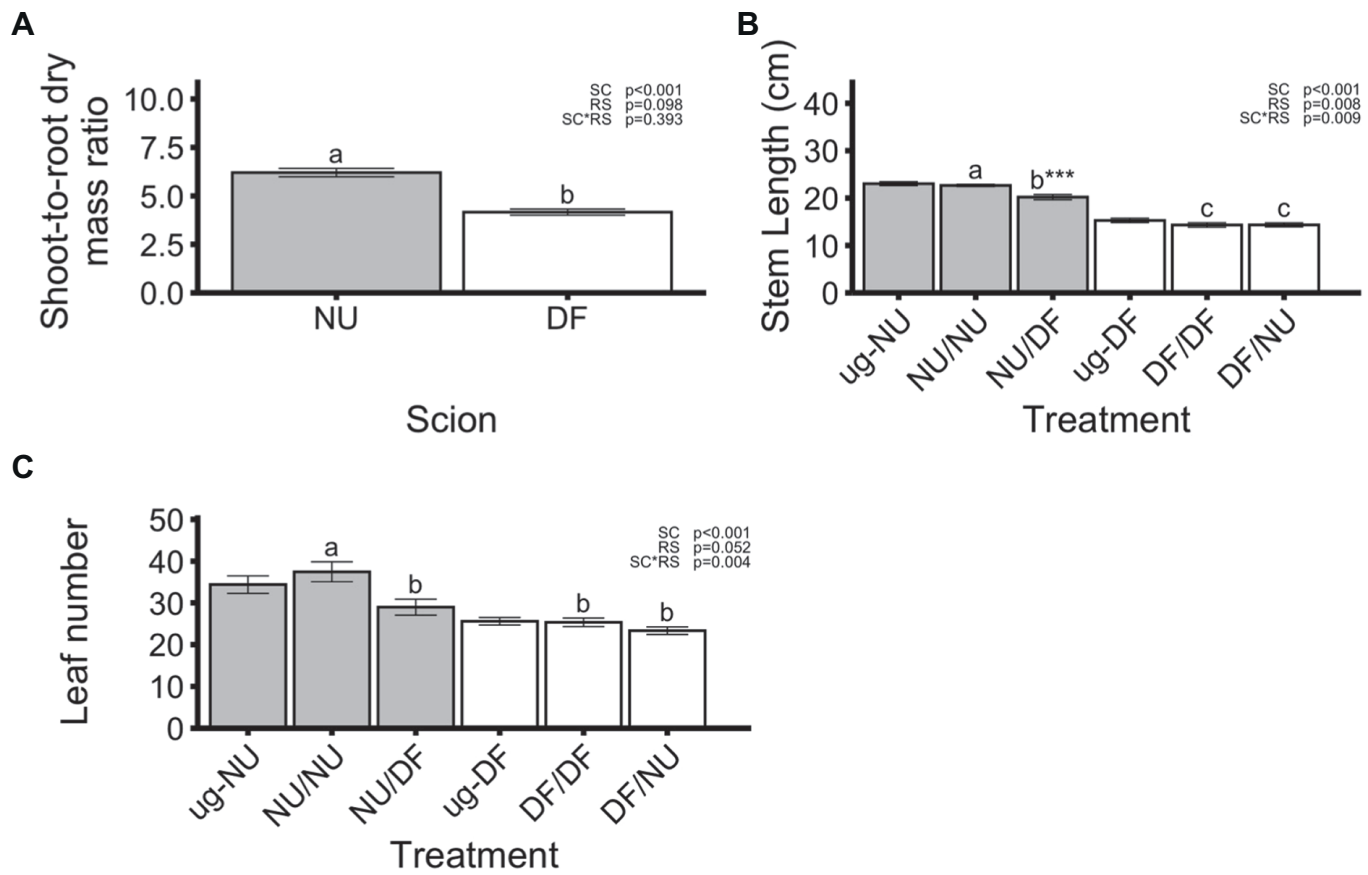
Shoot-to-root dry mass ratio **(A)** and main stem length **(B)** from both trials, and number of leaves, exceeding 2 cm, in the second **(C)** trial. Values are means of 24 **(A)**, 12 **(B)** or 6 **(C)** replicates for each scion **(A)** or treatment **(B,C)** and error bars represent standard error. Asterisks above bars indicate significant difference between grafted treatment and the un-grafted control of the same scion cultivar by Dunnett’s test [^***^(*p* ≤ 0.001), ^**^(*p* ≤ 0.01), and ^*^(*p* ≤ 0.05)]. *p*-values indicate significance of scion (SC), rootstock (RS) and scion by rootstock interaction (SCxRS) effects from the factorial analysis of self- and inter-cultivar grafted plants (four treatments excluding un-grafted controls). Letters indicate mean separations for the four grafted treatments by Tukey’s HSD (p ≤ 0.05) applied when SCxRS interaction was significant. Gray and white bars represent plants with NU and DF shoot systems, respectively.

In the first trial, dry root mass was significantly affected by scion genotype with NU scions producing root systems that were 21% greater than those produced by DF scions, regardless of the genotype of the rootstock (Figure 1C). However, neither self- nor inter-cultivar grafting significantly affected the dry root mass of either scion cultivar relative to the un-grafted controls (Figure 1C). In the second trial, there was a significant scion x rootstock interaction (Figure 1D). Dry root mass of self-grafted and inter-cultivar grafted plants was not significantly different within either scion cultivar. However, root dry mass for plants with NU root systems was reduced by 26% with DF scions (DF/NU) compared to self-grafted (NU/NU; Figure 1D). In contrast, dry root mass of plants with DF root systems were not different regardless of scion genotype (NU/DF vs. DF/DF). For NU scions, grafting to DF rootstocks (NU/DF) significantly reduced the root dry mass by 28%, relative to ug-NU, while self-grafting (NU/NU) had no effect (Figure 1D). Similarly, in DF scions, inter-cultivar grafting (DF/NU) resulted in 31% less root mass, compared to ug-DF. However, unlike NU scions, self-grafting DF (DFDF) also resulted in 17% less root mass, relative to ug-DF (Figure 1D). Despite interactions of scion and rootstock affecting shoot or root dry mass, the shoot-to-root dry mass ratio was only affected by the scion genotype with NU scions showing 49% higher ratios of shoot-to-root mass (Figure 2A). Additionally, when compared with un-grafted plants, neither self-grafting, nor inter-cultivar grafting affected the shoot-to-root ratio for either cultivar (Figure 2A).

Main stem length was significantly affected by the scion and rootstock genotype as well as the scion x rootstock interaction (Figure 2B). While both treatments with NU scions (NU/NU and NU/DF) had significantly longer main stem lengths, compared to the two treatments with DF scions (DF/DF and DF/NU), DF rootstocks reduced stem length for NU scions (NU/DF) by 11% compared to the self-grafted NU (NU/NU). However, self- (DF/DF) and inter-cultivar grafted DF (DF/NU) were not significantly different for stem length (Figure 2B). For NU scions, grafting to DF rootstock (NU/DF) significantly reduced main stem length by 12%, relative to ug-NU but self-grafting had no effect (Figure 2B). Main stem length was not significantly affected, relative to ug-DF, for self-grafted DF (DF/DF) or DF grafted to NU (DF/NU) rootstocks (Figure 2B).

Leaf number was not significantly affected by scion or rootstock genotype in the first trial (data not shown). In the second trial, the leaf number was significantly affected by scion genotype as well as scion by rootstock interaction (Figure 2C). While grafting NU scions to DF rootstocks (NU/DF) significantly reduced the leaf number by 23% compared to NU self-grafts (NU/NU), leaf number for self- (DF/DF) and inter-cultivar (DF/NU) grafted DF were not significantly different (Figure 2C). However, for both cultivars neither self-grafting nor inter-cultivar grafting altered leaf number compared to the un-grafted controls (Figure 2C).

### Leaf Tissue Mineral Nutrient Content

**Figure 3:**
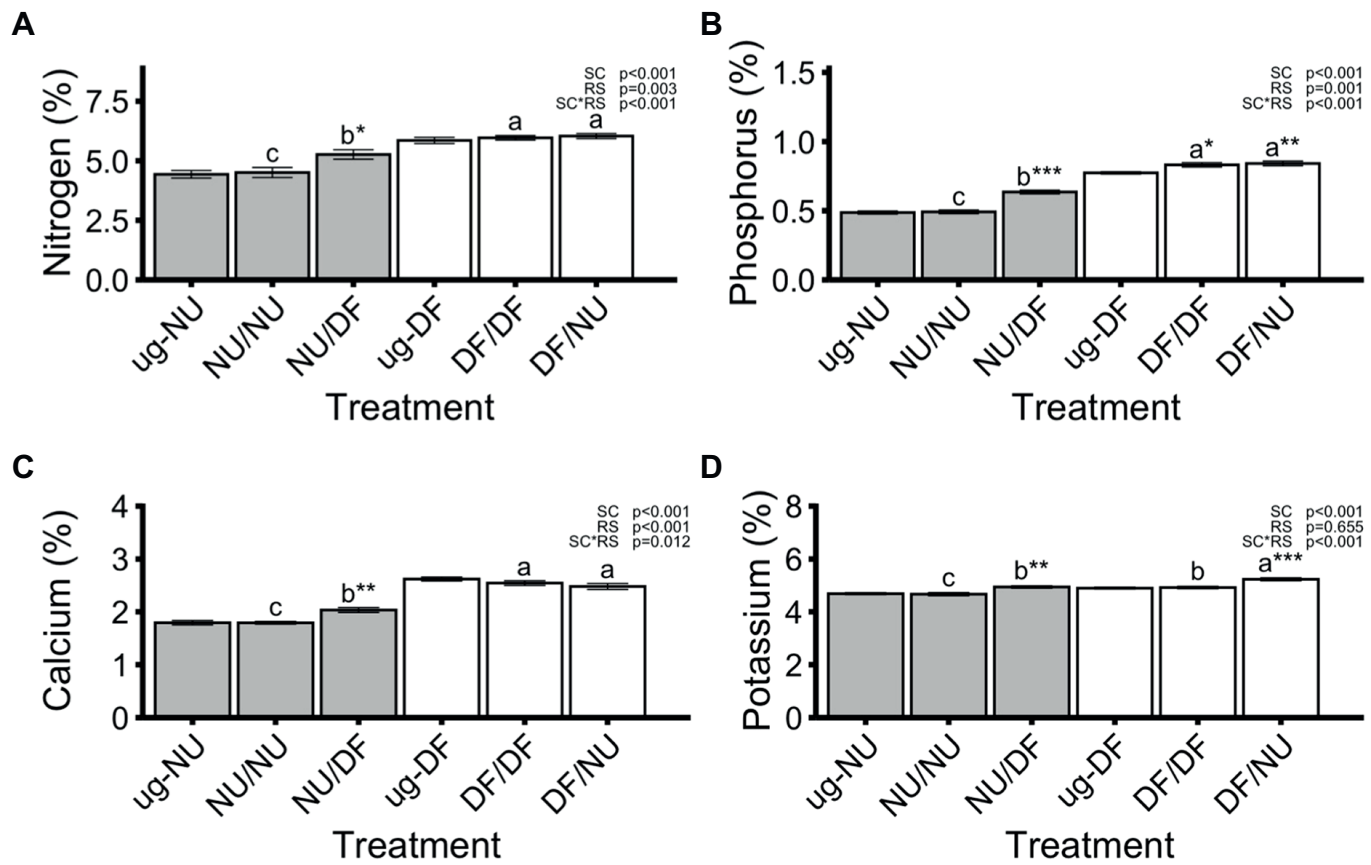
Concentrations of nitrogen **(A)**, phosphorus **(B)**, calcium **(C)**, and potassium **(D)** in dry shoot tissue in the second trial. Values are means of 6 replicates for each treatment and error bars represent standard error. Asterisks above bars indicate significant difference between grafted treatment and the un-grafted control of the same scion cultivar by Dunnett’s test [^***^(*p* ≤ 0.001), ^**^(*p* ≤ 0.01), and ^*^(*p* ≤ 0.05)]. *p*-values indicate significance of scion (SC), rootstock (RS) and scion by rootstock interaction (SCxRS) effects from the factorial analysis of self- and inter-cultivar grafted plants (four treatments excluding un-grafted controls). Letters indicate mean separations for the four grafted treatments by Tukey’s HSD (*p* ≤ 0.05) applied when SCxRS interaction was significant. Gray and white bars represent plants with NU and DF shoot systems, respectively.

Nitrogen (N), phosphorus (P), and calcium (Ca) concentrations in leaves were significantly affected by scion genotype, rootstock genotype and their interaction while potassium (K) was only affected scion genotype and scion by rootstock interaction (Figure 3). Grafting NU scions to DF rootstocks (NU/DF) significantly increased concentrations of N, P, Ca, and K by 17%, 29%, 13%, and 6%, respectively, compared to self-grafted NU (NU/NU) while grafting DF scions to NU rootstocks (DF/NU) did not alter N, P, or Ca concentration compared to DF self-grafts (DF/DF) but did significantly increase K concentration by 6% (Figure 3). Compared to ug-NU, N, P, Ca, and K concentrations significantly increased by 19%, 30%, 13% and 5%, respectively, for NU scions grafted to DF rootstocks (NU/DF) but were unaffected by self-grafting NU (NU/NU; Figure 3). For DF scions, N and Ca concentrations were unaffected by self- (DF/DF) or inter-cultivar grafting (DF/NU) relative to ug-DF (Figures 3A,C). However, grafting DF scions to NU rootstocks (DF/NU) significantly increased P and K concentrations, compared to ug-DF, by 9% and 7%, respectively (Figures 3B,D). Furthermore, self-grafting DF (DF/DF) significantly increased P concentration by 8% compared to ug-DF (Figure 3B).

**Figure 4:**
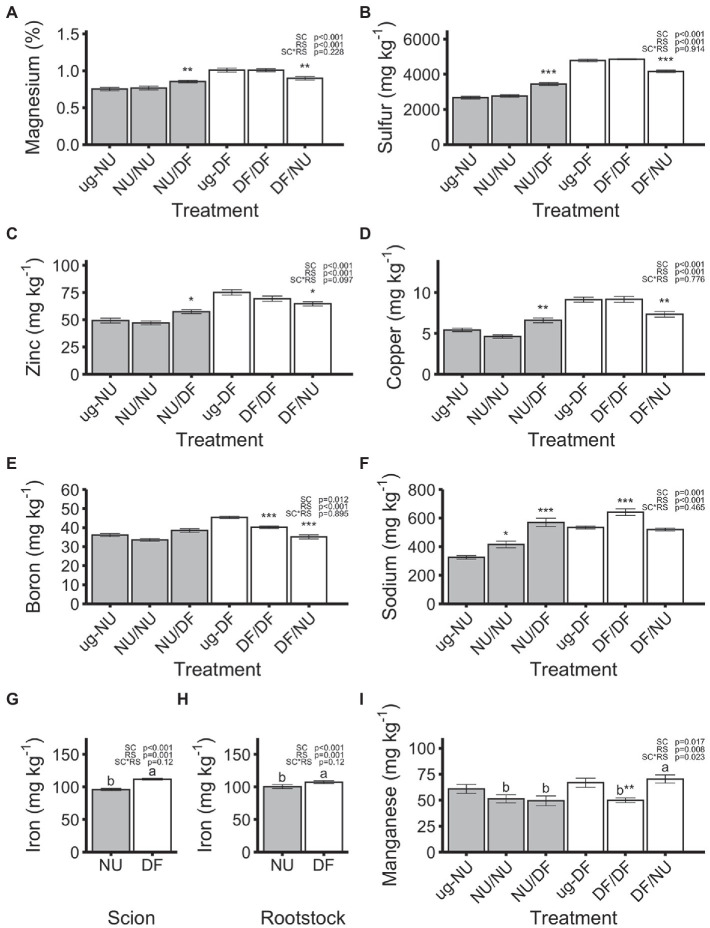
Concentrations of magnesium **(A)**, sulfur **(B)**, zinc **(C)**, copper **(D)**, boron **(E)**, sodium **(F)**, iron **(G,H)** and manganese **(H)** in dry shoot tissue in the second trial. Values are means of 6 replicates for each treatment **(A–F,I)** or 24 replicates for each scion or rootstock cultivar **(G,H)** and error bars represent standard error. Asterisks above bars indicate significant difference between grafted treatment and the un-grafted control of the same scion cultivar by Dunnett’s test [^***^(*p* ≤ 0.001), ^**^(*p* ≤ 0.01), and ^*^(*p* ≤ 0.05)]. *p*-values indicate significance of scion (SC), rootstock (RS) and scion by rootstock interaction (SCxRS) effects from the factorial analysis of self- and inter-cultivar grafted plants (four treatments excluding un-grafted controls). Letters indicate mean separations for the four grafted treatments by Tukey’s HSD (*p* ≤ 0.05) applied when SCxRS interaction was significant. Gray and white bars represent plants with NU and DF shoot systems, **(A–G,I)**, or root systems **(H)** respectively.

Magnesium (Mg), sulfur (S), zinc (Zn), copper (Cu), boron (B), sodium (Na), and iron (Fe) concentrations were not affected by the interaction of rootstock and scion cultivar but were affected by both scion and rootstock genotype (Figures 4A–H). Plants with DF scions (DF/DF and DF/NU) had 17%, 45%, 28%, 47%, 5%, 18%, and 17% higher concentrations of Mg, S, Zn, Cu, B, Na, and Fe, respectively, compared to plants with NU scions (NU/NU and NU/DF), regardless of rootstock (Figures 4A–G). Plants with DF rootstocks (DF/DF and NU/DF) had 12%, 20%, 14%, 32%, 15%, 29% and 7% higher concentrations of Mg, S, Zn, Cu, B, Na, and Fe, respectively, compared to plants with NU rootstocks (NU/NU and DF/NU), regardless of scion cultivar (Figures 4A–F,H). For plants with NU scions, grafting to DF rootstocks (NU/DF) significantly increased Mg, S, Zn, Cu, and Na concentrations by 13%, 29%, 17%, 22%, and 75%, respectively, relative to ug-NU, while self-grafting (NU/NU) did not alter Mg, S, Zn, and Cu concentrations but increased Na concentrations by 28% relative to ug-NU (Figures 4A–D,F). Additionally, neither self-grafting (NU/NU), nor grafting to DF rootstock (NU/DF) affected the concentration of B in NU scions (Figure 4E). Conversely, for DF scions, grafting to NU rootstock (DF/NU) significantly decreased Mg, S, Zn, and Cu concentrations by 11%, 13%, 14%, and 20%, respectively, compared to ug-DF (Figures 4A–D). Similarly, B concentration was significantly reduced, compared to ug-DF, by 11 and 23% due to self- (DF/DF) and inter-cultivar (DF/NU) grafting, respectively (Figure 4E). Na concentration in self-grafted DF (DF/DF) was significantly increased by 20% compared to ug-DF but unaffected by grafting to NU rootstock (DF/NU; Figure 4F). Iron concentrations were not significantly altered by either self- or inter-cultivar grafting for either cultivar relative to the un-grafted controls (Figures 4G,H). Manganese was significantly affected by scion genotype, rootstock genotype and their interaction (Figure 4I). Self- and inter-cultivar grafted plants with NU scions (NU/NU and NU/DF) were not significantly different. However, with DF scions, plants grafted to NU rootstocks (DF/NU) had 41% higher Mn concentrations than self-grafted plants (DF/DF). Additionally, while Mn concentration of NU scions was not altered, relative to ug-NU, Mn concentration in DF scions was significantly reduced, by 25% compared to ug-DF, with self-grafting but was not altered by grafting to NU rootstock.

## Discussion

The effect of grafting treatments on some plant growth parameters were similar but not identical in both trials. This could have been due to different environmental growing conditions, especially DLI which was almost twice as high during post grafting growth in trial 2 compared to trial 1. Walters and Currey (2018) showed that greater DLIs (7 vs. 15 mol m^−2^ d^−1^) increased growth of one of the cultivars used in this study (NU). Thus, while plants were harvested at similar growth stages in both trials, this difference could have contributed to the some of the differences in growth responses observed between the two trials.

Vegetable grafting is often practiced with the goal of improving plant vigor. However, in this study, grafting the less vigorous (DF) cultivar to the vigorous (NU) rootstock did not increase vigor or plant growth. Instead, grafting the vigorous (NU) cultivar to the less vigorous (DF) rootstock resulted in reduced growth relative to ug-NU, a response similar to the common practice of grafting to produce compact (dwarf) plant architecture in fruit trees. However, as leaf number was not significantly reduced and leaf size was not apparently different based on visual observation, we think that majority of biomass reduction could have come from the significantly shorter stem lengths. Basil plants are often sold as indoor containerized culinary herbs, and their morphological compactness is an important trait for aesthetic appearance (Currey et al., 2020). Compact architecture may be also beneficial for indoor vertical farms with limited head space. Non-chemical morphological control has been attempted through temperature control (Islam et al., 2016), brushing (mechanical stress; Appling, 2012), moisture control (Currey et al., 2019) and nutrient management (Currey et al., 2020). In fact, grafting fine-leaved bush basil onto a proprietary rootstock has been commercialized in Israel for their compact topiary-like morphology (Hishtil Ltd., Ashkelon, Israel). While additional research is required to confirm, grafting could be used to create shorter stature plants without reducing leaf yield.

The observed lack of vigor promotion in less vigorous (DF) scions grafted to vigorous (NU) rootstocks conflicts with reports for other species where increased vigor was found with grafting to vigorous cultivars as rootstocks under non-growth limiting conditions in watermelon (e.g., Oztekin et al., 2011; Bekhradi et al., 2012), tomato (*S. lycopersicum*), and eggplant (*S. melongena*; Leonardi and Giuffrida, 2006; Masterson et al., 2016). However, there have been similar cases where certain vigorous rootstocks have failed to promote vigor/yield in certain scions (Khah et al., 2006; Leonardi and Giuffrida, 2006; Bekhradi et al., 2012) or have even resulted in decreased vigor/yield (Kacjan-Maršić and Osvald, 2004; Suchoff et al., 2019) in other crop species grown under non-growth limiting conditions. Previous studies have also highlighted the importance of the scion-rootstock interaction in determining vigor in grafted plants (Kacjan-Maršić and Osvald, 2004; Leonardi and Giuffrida, 2006). Given the huge range of phenotypes of sweet basil (Simon et al., 1999), other combinations of scion and rootstock should be further investigated for basil.

For both basil scion cultivars, there was a significant reduction in root mass for inter-cultivar grafts (NU/DF and DF/NU) relative to their self-grafts (second trial), highlighting the scion-rootstock genotype interaction on root mass. In soybean (*Glycine max*), the ability of scions to alter root mass and architecture has been shown to be independent of the photosynthetic rate of the scion (Du et al., 2019). Other studies have suggested basipetal transport of hormones and proteins influence both root mass and root size (Spiegelman et al., 2015; McAdam et al., 2016). Interestingly, the root-to-shoot ratio was only affected by scion cultivar. This suggests that root mass may not be the critical factor determining shoot growth in basil. This conflicts with previous research that found increased shoot mass and root mass in basil inoculated with *Gigaspora rosea* (Copetta et al., 2006). However, in the present study water and nutrients were supplied regularly which could explain these differences. This also indicates root mass and therefore rootstock may play a role in conditions where water and nutrient supply limit plant growth. Nevertheless, it is possible that other characteristics including root architecture of the rootstocks that were not quantified in this study may have influenced the shoot growth. Root system size and architecture are often cited as one of the reasons for high performance of grafted plants. Vigorous vegetable rootstocks have been shown to have longer root length, smaller root diameter and higher specific root length (Suchoff et al., 2018) and increased root volume, surface area and branching (Huang et al., 2016; Liang et al., 2021). Conversely, dwarfing apple rootstocks have been shown to produce fewer fine roots than more vigorous rootstocks, indicating a role of root size in dwarfed growth (An et al., 2017).

The previously mentioned root physical traits as well as improved nutrient transporters (Albornoz et al., 2018) are thought to enhance water and nutrient uptake in grafted plants leading to improved plant vigor. However, our results showed that grafted basil accumulated nutrients in shoot tissue without a corresponding increase in growth. This agrees with previous research on nutrient requirements in un-grafted basil grown in protected hydroponic or substrate culture. For example, Walters and Currey (2018) found that increasing overall nutrient solution concentrations increased tissue nutrient content but not plant growth of one of the cultivars used in the present study (NU). Similarly, Currey et al. (2020) found that shoot dry mass and height of container-grown basil increased with increasing P supply up to 20 mg l^−1^ but observed no additional increases at higher supply despite continued linear increase in P and N concentrations in plant tissue. Using the same cultivars as this study (DF and NU), Gillespie et al. (2020) found that reduced uptake of P, Ca, Mg, S, B, Mn, and Zn under low nutrient solution pH had no significant effect on the basil plant growth. The lack of response to increased nutrient supply (observed in this and previous studies) contrasts with other studies of open field basil increasing yield with increased applications of fertilizers (Zheljazkov et al., 2008; Pandey et al., 2016). This difference could be due to the regular supply of water and nutrients in the present study as opposed to open field basil production where these factors may limit plant growth. Thus, differences in root system traits may not have played a critical role in plant growth. Our finding also differs from grafting studies in tomato (Djidonou et al., 2019) and cucurbits (Nawaz et al., 2017; Liang et al., 2021) that have found increased plant vigor and yield due to the enhanced nutrient uptake and supply by the rootstock. While basil growth seems relatively unaffected by nutrient supply, studies have shown nutrient supply to alter the concentrations of commercially important compounds in basil (Zheljazkov et al., 2008; Ciriello et al., 2020; Kolega et al., 2020). The altered concentrations of nutrients such as N and S in inter-cultivar grafts suggests that essential oil composition in basil cultivars could be affected by grafting particular scions and rootstocks, although we did not examine such alterations in the present study. Given the apparent “insensitivity” of basil growth to increased nutrient concentration observed in this and previous studies, the other factors may have influenced grafted basil plant growth in the present study. For example, long distance movement nucleic acids across the graft union as well as associated epigenetics mechanisms like DNA methylation/demethylation, histone modification and RNA mediated actions have been documented in grafted plants and can influence the phenotype (Tsaballa et al., 2021). Additionally, phytohormones like cytokinin (Yamasaki et al., 1994; Ghanem et al., 2011; Feng et al., 2017; Martínez-Andújar et al., 2017; Nawaz et al., 2017), gibberellins (Wang et al., 2012; Regenault et al., 2015; Tworkoski and Fazio, 2016), and ethylene precursors (Martínez-Andújar et al., 2017) have also been shown to affect the growth of grafted plants. Thus, future studies in grafted sweet basil should examine these alternate mechanisms for altered plant growth.

Another benefit of grafting is the rootstock capacity to exclude or sequester of unwelcome or toxic mineral elements. The lower Na concentration in plants with NU rootstocks compared to plants with DF rootstocks may represent differing strategies for tolerating excess Na. Thus, the drastic (75%) increase in Na concentration observed in NU scions grafted to DF rootstocks compared to ug-NU could be a result of decreased exclusion and/or retention of Na by the DF root system. Differing Na exclusion strategies have been found in cucumber and pumpkin reciprocal grafts; cucumber root systems did not exclude Na while pumpkin roots reduced Na uptake by 50% leading to lower growth reduction for cucumber grafted to pumpkin than pumpkin grafted to cucumber relative to self-grafted plants (Huang et al., 2013). Basil is considered relatively salt tolerant due to high expression of catalase and retention of Na in roots and stems (Tanaka et al., 2018). Additionally, no visual indications of Na toxicity were observed. Therefore, although differences in Na tissue concentration are striking, it is unlikely that high levels of sodium or nutrients would cause the growth reduction seen in NU/DF.

Previous research suggests that self-grafted plants generally respond similarly to un-grafted plants for some crop species. For example, Oztekin et al. (2011) found self- and un-grafted watermelon did not differ in stem length, shoot mass, and root mass. Similarly, a meta-analysis of published tomato grafting projects found that yield of self- and un-grafted plants only differed in 6% of cases, but could occasionally be dramatic (Grieneisen et al., 2018). We found self- and un-grafted plants of both cultivars were similar for most growth parameters. However, shoot mass was reduced for NU/NU in the first trial and shoot and root mass were reduced for DF/DF in the second trial compared to their respective controls. Similarly, while most mineral concentrations were unaffected by self-grafting, P and Na concentrations increased with self-grafting in DF and both cultivars, respectively, and B and Mn concentrations decreased with self-grafting in DF. This agrees with previous studies in solanaceous crops that found few if any differences in mineral concentration between self- and un-grafted plants (Sánchez-Rodríguez et al., 2014; Kumar et al., 2015; Sánchez-Torres et al., 2015). While it unlikely that the healed graft union presents a barrier to water or nutrients in many plants (Gautier et al., 2019) it is possible that the differences in growth and nutrient content observed in this study could be due to a stress response triggered by the wounding and healing process used, which has not been subjected to the same optimization as grafting and healing processes for commercially grafted crops. For example, Khah et al. (2006) observed higher early season yields in un-grafted tomato relative to self-grafted and suggested that this could be due to a lack of stress imposed by grafting/healing. However, stress during graft healing alone would not explain why Mn concentrations are lower than ug-DF in DF/DF but not DF/NU, as both were exposed to the same conditions.

## Conclusion

In this first report of basil grafting effects on growth and mineral nutrient concentrations, we showed that two cultivars of basil, NU and DF, could be reciprocally grafted successfully. We also showed that grafted basil growth differed from un-grafted control plants. However, unlike in other crops, grafting the less vigorous cultivar (DF) to the more vigorous rootstock (NU) did not increase growth parameters and in general decreased nutrient concentrations in the leaves. Additionally, grafting the vigorous NU scions to less vigorous (DF) rootstocks generally resulted in a reduced plant growth and increased leaf tissue nutrient concentrations. Based on these findings and other research it seems that basil is relatively insensitive to nutrient concentrations, and thus changes in basil growth due to grafting are likely due to other factors that were outside the scope of the present study rather than nutrient uptake. Additional research is required to determine specific mechanisms influencing growth and nutrient uptake in grafted basil.

## Data Availability Statement

The datasets obtained from this study can be found in the Controlled Environment Agriculture Open Data (https://ceaod.github.io/).

## Author Contributions

JRH contributed to conception and design of the study, data collection, data analysis, and writing of the manuscript. CK contributed to the conception and design of the study, data analysis, and writing of the manuscript. All authors contributed to the article and approved the submitted version.

## Funding

This project was supported by USDA NIFA Specialty Crop Research Initiative Grants program (Project number: 2016-51181-25404).

## Conflict of Interest

The authors declare that the research was conducted in the absence of any commercial or financial relationships that could be construed as a potential conflict of interest.

## Publisher’s Note

All claims expressed in this article are solely those of the authors and do not necessarily represent those of their affiliated organizations, or those of the publisher, the editors and the reviewers. Any product that may be evaluated in this article, or claim that may be made by its manufacturer, is not guaranteed or endorsed by the publisher.
